# Hybrid knowledge transfer for MARL based on action advising and experience sharing

**DOI:** 10.3389/fnbot.2024.1364587

**Published:** 2024-05-07

**Authors:** Feng Liu, Dongqi Li, Jian Gao

**Affiliations:** ^1^School of Marine Science and Technology, Northwestern Polytechnical University, Xi'an, China; ^2^Kunming Precision Machinery Research Institute, Kunming, China

**Keywords:** knowledge transfer, marl, experience sharing, action advising, multi-agent systems

## Abstract

Multiagent Reinforcement Learning (MARL) has been well adopted due to its exceptional ability to solve multiagent decision-making problems. To further enhance learning efficiency, knowledge transfer algorithms have been developed, among which experience-sharing-based and action-advising-based transfer strategies share the mainstream. However, it is notable that, although there exist many successful applications of both strategies, they are not flawless. For the long-developed action-advising-based methods (namely KT-AA, short for knowledge transfer based on action advising), their data efficiency and scalability are not satisfactory. As for the newly proposed experience-sharing-based knowledge transfer methods (KT-ES), although the shortcomings of KT-AA have been partially overcome, they are incompetent to correct specific bad decisions in the later learning stage. To leverage the superiority of both KT-AA and KT-ES, this study proposes KT-Hybrid, a hybrid knowledge transfer approach. In the early learning phase, KT-ES methods are employed, expecting better data efficiency from KT-ES to enhance the policy to a basic level as soon as possible. Later, we focus on correcting specific errors made by the basic policy, trying to use KT-AA methods to further improve the performance. Simulations demonstrate that the proposed KT-Hybrid outperforms well-received action-advising- and experience-sharing-based methods.

## 1 Introduction

Reinforcement Learning (RL) has been developed for decades, achieving remarkable advances in chess (Silver et al., 2018), video games (Ye et al., 2020), smart grids (Chen et al., 2022), robotic control (Ibarz et al., 2021), and even the control of tokamak plasmas (Degrave et al., 2022). Among these achievements, researchers have also developed theories and algorithms for Multi-Agent Reinforcement Learning (MARL), an important branch of RL, playing a notable role in recent years in solving decision-making problems of Multi-Agent System (MAS). However, in contrast to RL with a single agent, the difficulty in decision-making for MARL increases significantly due to the growing number of agents. As a result, it is necessary to study how to accelerate the learning process of MARL (Wang et al., 2021).

Knowledge Transfer (KT) is one of the effective approaches to enhance the learning speed of MARL (Ilhan et al., 2021). As a type of Transfer Learning (TL) method, KT focuses on transferring the knowledge of one agent to others in a MAS. Starting from Episode Sharing, which intuitively shares successful trajectories in episodic tasks, KT has been applied in various multi-agent tasks (Tan, 1993). Action-Advising-based Knowledge Transfer (KT-AA) is one of the most popular KT methods. Instead of transferring whole trajectories, agents with KT-AA only share actions in specific circumstances, evidently reducing the load of communication channels (Silva et al., 2020). While KT-AA brings benefits in communication, it also makes it critical to address the when-to-transfer issue. To this end, Chernova and Veloso (2009) proposed a confidence-based trigger condition to determine whether an agent is familiar with a certain state, estimating the quality of decision-making. Once an agent is predicted to make bad or uncertain decisions, it acts as an advisee and transfers its current state observation to the advisor. Then, the advisor will make a decision for the advisee, selecting and sending back a better action to the advisee. Although Chernova and Veloso (2009) did pioneering work and designed the basic action-advising framework, their study still has space for improvement. To reduce communication, Torrey and Taylor (2013) proposed to teach on a budget, in which the advisors send action advice to the advisees with a limited amount of advice. Benefiting from the delicately designed trigger conditions to determine when an advisor should teach, this transfer approach can get satisfactory performance with a significantly lower communication load. Later, Amir et al. (2016) extended the teaching-on-a-budget framework, introducing a kind of hybrid trigger condition by considering both the advisors' monitoring and the advisees' requirements.

Another important branch of KT-AA is to explore its potential in learning-from-scratch scenarios. Most of the above studies assume that there is at least one moderate-level expert in the system, while for the learning-from-scratch scenarios, all the agents learn simultaneously from random policies. In these circumstances, one can not define a fixed advisor before the learning process starts, making it harder to design trigger conditions. Silva et al. (2017) modified and applied the teaching-on-a-budget framework to the learning-from-scratch scenarios. Unlike the above algorithms in which the advising agents are fixed, this study requires all agents to perform as both advisors and advisees. The role of an agent will be determined by two sets of metrics, which take state familiarity and Q values into consideration. Hou et al. (2021) introduced memetic computation into the MARL system and proposed an Evolutionary Transfer Learning (eTL) method. By modeling the learning agents as memetic automatons, eTL provides two metrics to evaluate whether an agent has learned a better policy than the others in the training process, forming a dynamic indicator as the trigger condition to provide action advice. Moreover, eTL also employed stochastic mutation, an important operator in memetic computing, in the KT process, further enhancing the performance.

While KT-AA has primarily shown its ability in learning-from-scratch scenarios, it faces two main challenges. The first challenge arises when the number of agents grows, causing an exponential increase in the computation and communication load, which indicates that it is hard to scale (Wang et al., 2021). The other challenge lies in the design of trigger conditions (Omidshafiei et al., 2019). Without any agent possessing an assured decision-making ability, we should reconsider the qualification standard of action. In previous studies with expert-level agents, the goal of KT was to select actions with better expectations. But for the learning-from-scratch scenes, there is nothing to rely on to predict the potential consequences of an action. Thus, the researchers have focused on the primary goal of KT, which is to boost the learning speed Wang et al. (2024).

Recently, Experience-Sharing-based Knowledge Transfer (KT-ES) has been proposed to tackle these problems. Wang et al. (2022) extended the concept of memetic knowledge transfer, developing a KT-ES algorithm, namely, MeTL-ES. Inspired by implicit meme transmission, MeTL-ES proposed to share specific experiences, i.e., state transition tuples, rather than specific actions, among the learning agents. This mechanism converts the bi-directional information flow in each transfer to a one-way manner, which solves the scalability issue by nature. Moreover, MeTL-ES employs a new idea in designing the trigger condition. Rather than selecting experiences with higher rewards or Q values, it shares experiences with stochastic rules. Specifically, in the early learning stage, agents using MeTL-ES share most randomly, which matches the need for exploration in RL. When the performance is enhanced to a certain level, metrics like Q values get more accurate, and the exploration is roughly enough; MeTL-ES tends to indicate the value of an experience via Q values. Only experiences with higher possible outcomes will get transferred. Using this mechanism, we can observe a rapid rise in performance in the early stage, which benefits from the sufficient exploration brought by MeTL-ES.

However, KT-ES methods such as MeTL-ES lack the focus on the later stage, which may hinder the performance of KT-ES, indicating that KT-AA and KT-ES have complementary features. Out of this consideration, this study proposes a Hybrid Knowledge Transfer method, KT-Hybrid, by binding the advantage of KT-AA and KT-ES, expecting to promote the learning performance of the randomly initialized agents in the whole process. Overall, the contributions of this study are 3-fold:

1. This study discusses the scopes of application of KT-AA and KT-ES and presents a novel two-phase knowledge transfer framework to enhance the learning speed of MARL accordingly;
2. Based on the unique features of the framework, a novel algorithm, KT-Hybrid, is proposed, along with the corresponding trigger conditions to balance exploration and exploitation;
3. Building on the well-received Minefield Navigation Tasks, empirical studies in several typical scenarios are provided in this study, indicating that the proposed KT-Hybrid outperforms popular KT-AA and KT-ES algorithms.

## 2 Preliminaries

This section introduces some basic concepts and knowledge relevant to this study.

### 2.1 RL and MDP

RL is an effective way to solve decision-making problems that can be modeled as Markov Decision Processes (MDP), and the entity that makes decisions to achieve certain tasks in a given environment is called an agent (Barto et al., 1989; Sutton and Barto, 2018). Typically, an MDP can be described by a 5-tuple, 〈S,A,T,R,γ〉, in which $S\to {\mathbb{R}}^{S}$ denotes the *S*-dimensional state space of the environment. $A=\left\{a_{1},a_{2},\cdots \;,a_{K}\right\}\to {\mathbb{R}}^{K}$ represents the *K*-dimensional action space of the agent, i.e., all the *K* actions that an agent can take. $T\left(s,a,s^{\prime }\right):S\times A\times S\to \left[0,1\right]$ works as the state transition function, providing the probability that the current state *s* will transfer to *s*′ when the agent takes action *a*. R(s,a):S×A→ℝ is the reward function. The discount factor γ defines how a future reward will be discounted.

Given the definition of an MDP, the experience of an agent can be defined as 〈*s, a, r, s*′〉, in which *s* denotes the state of the environment, *a* represents the action that the agent takes, *r* shows the reward that the agent can earn from the environment when implying action *a*, and *s*′ informs the resultant environment state that corresponds to *s* and *a*.

Generally, the goal of an RL agent is to learn a policy π:S×A→[0,1] to conduct sequential decision-making processes in the MDP. Ideally, the policy will gradually converge to an optimal policy, π^*^, that can maximize the state value of the initial state. The state-value function can be given as follows:


(1) $$\begin{array}{l} v_{\pi }\left(s\right)={𝔼}_{\pi }\left[\overset{\infty }{\underset{k=0}{\sum }}\gamma^{k}r_{t+k+1}|s_{t}=s\right], \end{array}$$

in which *s*_*t*_ and *r*_*t*_ imply the state and reward at time *t*.

Q-learning is one of the most popular RL algorithms (Watkins and Dayan, 1992), which learns to estimate the Q-value function given as follows:


(2) $$\begin{array}{l} Q_{\pi }\left(s,a\right)={𝔼}_{\pi }\left[\overset{\infty }{\underset{k=0}{\sum }}\gamma^{k}r_{t+k+1}|s_{t}=s,a_{t}=a\right]. \end{array}$$

Moreover, the update rule can be written as follows:


(3) $$\begin{array}{l} Q_{\pi }\left(s,a\right)=Q_{\pi }\left(s,a\right)+\alpha \left(y-\right), \end{array}$$

in which $y=r+\gamma \underset{a^{\prime }}{\max}Q\left(s^{\prime },a^{\prime }\right)$ denotes the target. Now, the TD error can be given as follows:


(4) $$\begin{array}{l} \delta =y-Q\left(s,a\right). \end{array}$$

Since Deep Neural Networks (DNN) have been developed greatly in the past decade, Mnih et al. (2013) and Mnih et al. (2015) proposed Deep Q-Network (DQN), an incorporation of DNN and Q-learning. Parameterized by vector θ, the loss function of the neural network in the learning process can be given as follows:


(5) $$\begin{array}{l} L\left(\theta \right)={𝔼}_{s,a,r,s^{\prime }\sim D}\left[{\left(y^{-}-Q\left(s,a;\theta \right)\right)}^{2}\right], \end{array}$$

where D is the experience buffer, which is designed to break the correlations of the data. Another part that can do the same is the introduction of the target network. In short, the target network is a copy of the policy network whose parameters are updated intermittently, providing the target value *y*^−^. Denoting the parameters of the target network as θ^−^, the target value can be calculated as follows:


(6) $$\begin{array}{l} y^{-}=r+\gamma \underset{a^{\prime }}{\max}Q\left(s^{\prime },a^{\prime };\theta^{-}\right). \end{array}$$

### 2.2 MARL and SG

When the number of entities that need to make decisions extends from one to several, the underlying model will be extended from MDP to Stochastic Games (SG) (Shapley, 1953). An SG can be described by the tuple of 〈n,S,A,T,R,γ〉, in which *n* is the number of agents and S represents the state of the environment. $A:A_{1},A_{2},\cdots \;,A_{n}$ denotes the joint action space, where $A_{i}$ gives the action space of agent *i*. In this setting, we can define the action of agent *i* as $a_{i}\in A_{i}$ and the joint action a∈A as the joint action of all the agents. Then, we can write the state transition function T as $T\left(s,a,s^{\prime }\right):S\times A\times S\to \left[0,1\right]$.

Typically, in multi-agent tasks, the agents cannot have access to the global state of the environment, i.e., s∈S. In contrast, they will have different observation functions, denoting $O_{i}$ for each agent *i*. Now, the tuple of an SG can be extended to 〈n,S,O,O,A,T,R,γ〉, in which the added $O:O_{1}\times O_{2},\cdots \;,O_{n}$ describes the joint observation space of the agents and *O*:*O*_1_×*O*_2_⋯ × *O*_*n*_ gives the observation functions. In this study, we only consider the commonly used homogeneous multi-agent systems in which the agents share the same observation function *O*.

In this circumstance, conventional single-agent RL is not capable of handling such tasks, and the community has its sights set on Multi-agent Reinforcement Learning (MARL). Tan (1993) proposed to apply single-agent Q-learning to multi-agent task scenarios, forming the Independent Q-Learning (IQL). The learning process of IQL requires each agent to act and collect experience independently. On this basis, the agents will also learn independently according to their own rollouts. It is an intuitive but effective way to solve multi-agent tasks using RL. In the era of deep learning, Tampuu et al. (2017) integrated DNN and advanced techniques such as experience replay to IQL and formalized the Independent DQN (I-DQN), promoting the learning performance to a higher level. By combining the classic learning algorithm and newly developed techniques, I-DQN has inevitable advantages in scalability and flexibility (Foerster et al., 2017). Thus, we adopt I-DQN as the basic learning algorithm in this study.

### 2.3 Knowledge transfer for independent MARL

Knowledge transfer mechanisms aim to leverage insights gained by one agent to accelerate learning or improve performance for another agent. This can be particularly beneficial in MARL since the learning agents are able to share various types of useful information.

For I-DQN-like independent MARL algorithms, there are two popular branches of knowledge transfer, which are KT-AA and KT-ES. In KT-AA, agents share action advice with each other. Specifically, a learning agent with KT-AA will determine dynamically whether to ask for advice during the learning process (Torrey and Taylor, 2013). Once the ask process is triggered, the agent will send the current state, and it encounters to the other agents for help, and the agents receiving the query will provide a potential action. The most distinctive advantage of KT-AA lies in the direct correction of a specific action, but at the same time, it suffers from bad scalability due to its bi-directional information exchange. KT-ES is a recently developed knowledge transfer approach. Compared with KT-AA, which was initially developed for learning systems with experts, KT-ES was proposed for simultaneously learning scenarios (Wang et al., 2022). Given this background, KT-ES focuses more on the long-term benefits by encouraging exploration, the rationality of which has also been validated recently in KT-AA (Wang et al., 2024). To achieve this, agents using KT-ES methods share personal experiences in a one-way manner. Typically, the experiences are defined by the state transitions together with variables used to calculate the sharing trigger conditions. The stochastic sharing without consideration of specific states leads to unfamiliar situations for which exploration may emerge.

To summarize, it is clear that both KT-AA and KT-ES can enhance learning performance, but with different motivations. In the following section, we will introduce a novel knowledge transfer method that combines the strengths of KT-AA and KT-ES to enhance the learning performance of independent MARL systems further.

## 3 KT-Hybrid

This section introduces the overall architecture of the proposed KT-Hybrid algorithm, along with the design details answering the questions of what to transfer, when to transfer, and how to use the received knowledge.

### 3.1 Architecture

Recalling the goal that we require the knowledge transfer to achieve. Primarily, we need the agents in MARL systems to learn as fast as possible, at which period the agents will form basic-level policies that can roughly obtain a satisfactory performance. Meanwhile, to ensure the learning speed, i.e., the speed to form a basic performance, it is not ideal to put too much extra computational load on the agents at this early learning stage. In addition, to avoid possible obstacles to large-scale MARL, it would be better to adopt knowledge transfer methods with guaranteed scalability. These discussions make the KT-ES a great solution for the MARL problems, especially for the early learning stage.

While KT-ES meets the needs in the early learning stages, it usually has weaker performance in the late learning stage. The main reason for this problem is that the KT-ES methods give much more look to the data efficiency and scalability rather than the decision-making quality of any specific actions. Thus, agents with KT-ES tend to have a roughly qualified policy in a very short time, but the performance may remain fixed or even drop slightly in the late stage. This phenomenon also matches with both the results reported in the study by Wang et al. (2022) and our pilot study.

On the contrary, KT-AA focuses on every single decision-making performance of an agent at the cost of scalability. In specific, KT-AA needs bidirectional interactive communication rather than unidirectional broadcast-like communication in KT-ES. However, this brings about the unique advantage of KT-AA and KT-AA can help the agents in specific decision-making steps. By incorporating KT-AA, every possible action of each agent has some possibility of getting double-checked by other agents, helping to prevent bad decisions in specific states. This feature of KT-AA indicates that although it may be inefficient in the early learning stage, it has the potential to further enhance the policies that are roughly trained. In summary, KT-ES and KT-AA exhibit distinct advantages across different learning stages, suggesting the potential for improved learning performance through their combination.

**Figure 1:**
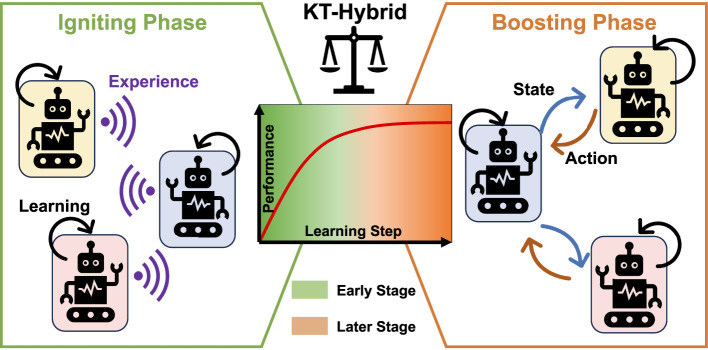
Overall architecture of KT-Hybrid. Robots in different backgrounds represent different learning agents, purple waves show the shared experience in the Igniting Phase, black arrows depict the self-learning process of the agents, and the blue and orange arrows are the transferred state and action in the Boosting Phase, respectively. In the central subfigure, the green background shows the early learning stage, and the orange shows the later learning stage. These two stages are divided with a blurred line, indicating that the shift timing should be selected by balancing the exploration and exploitation of these two stages.

Building on the above discussions, the goal of this study is to design hybridization of KT-ES and KT-AA, KT-Hybrid, trying to make full use of their complementary features. Overall, the KT-Hybrid follows a two-phase structure, as shown in Figure 1.

In the MARL process, the first phase is called the Igniting Phase, which aims to rapidly learn a moderate-level policy in the early stage. Thus, we design experience-sharing-based knowledge transfer mechanisms in the Igniting Phase, leveraging the benefits of KT-ES in terms of fast learning speed and high data efficiency to provide the users with workable policies as soon as possible.

While for the learning process in the late stage, the Boosting Phase takes over to further boost the policy to a higher level with more communication effort. At this stage, the requirement of the user is converted from getting workable policies to tuning the policies for better performance. Therefore, we need to follow the principles of the action-advising-based knowledge transfer approaches in the Boosting Phase, expecting to obtain better performance even with a higher load.

Since the primary goal of knowledge transfer is to promote learning speed, it is unacceptable for the KT-Hybrid if the transfer scheme is of high complexity order, which may lead to significant computational costs.

Now, it is obvious that due to the complex mechanism, the performance of KT-Hybrid is promising, yet the design will be challenging. Given this framework, we will detail the format of knowledge in the two phases in Section 3.2, introduce a novel trigger condition to balance the two phases in Section 3.3, and design the learning scheme accordingly in Section 3.4.

### 3.2 What to transfer

This subsection will introduce what type of knowledge is transferred in KT-Hybrid. To meet the two-phase architecture of KT-Hybrid, we need to design the form of knowledge separately.

For the Igniting Phase, agents are required to share implicit knowledge that works as ingredients in the learning process, i.e., experiences (Wang et al., 2022), rather than explicit actions, for fast promotion of the policies. Formally, for any agent *i* at time *t*, this agent will observe the state of the environment and obtain its own observation at this time $o_{t}^{i}$. Then, agent *i* makes a decision to take action $a_{t}^{i}$ according to $o_{t}^{i}$ and its current policy $\pi_{t}^{i}$. Then, the state of the environment *s* will transit to a new state *s*′, and agent *i* will get an updated state observation, denoting as $o_{t+1}^{i}$. Meanwhile, agent *i* will also get the reward signal $r_{t}^{i}$ from the environment. Now, after a complete state (observation) transition, the experience of agent *i* at time *t* can be formulated as follows:


(7) $$\begin{array}{l} E_{t}^{i}=\langle o_{t}^{i},a_{t}^{i},r_{t}^{i},o_{t+1}^{i}\rangle . \end{array}$$

Then, agent *i* will assess the quality of $E_{t}^{i}$; once $E_{t}^{i}$ is suitable for sharing, it will be broadcast as a knowledge package $K_{t}^{i}$ by agent *i* to the other agents for further learning. Formally, the knowledge at time *t* in the Igniting Phase, i.e., $K_{t}^{I}$, can be expressed ad follows:


(8) $$\begin{array}{l} K_{t}^{I}=\left\{K_{t}^{i}|i=1,\cdots \;,n\right\}=\left\{pI_{t}^{i}E_{t}^{i}|i=1,\cdots \;,n\right\}. \end{array}$$

In the knowledge shown in Equation 8, $pI_{t}^{i}$ denotes the probability for agent *i* to share the current experience in the Igniting Phase. Detailed description and definition of $pI_{t}^{i}$ will be provided in Section 3.3. In addition, the way how KT-Hybrid uses the shared knowledge $K_{t}^{I}$ for learning will be described in Section 3.4.

The benefits of defining the state (observation) transition as the carrier of knowledge are threefold. First, sharing transitions that an agent has just experienced does not need any extra computation or memory storage, which means that the agents can learn faster with no extra loads. Second, since the broadcasting of experiences is a one-way communication, when the number of agents grows, the overall communication load will only increase linearly. This results in a good scalability. Finally, the state transition is commonly used as experience in RL, which makes KT-Hybrid a general knowledge transfer method for a wide range of MARL algorithms.

As learning proceeds, KT-Hybrid will turn to the Boosting Phase. At this phase, the agents will have moderate-level policies to handle the task in the environment. Thus, it is time for the agents to transfer explicit knowledge, i.e., the actions, to each other. For any agent *i*, to get action advice for observation $o_{t}^{i}$ at time *t*, it will query the other agents with the observation $o_{t}^{i}$. Once receiving the query, other agents will provide actions according to their own policies. Take agent *j* as an example. At time *t*, it generates actions $a_{t}^{ij}$ according to $\pi_{t}^{j}$ and $o_{t}^{i}$, in which $a_{t}^{ij}$ represents the action generated by agent *j* to solve the observation of agent *i* at time *t*. Then, for agent *i*, it will receive a collection containing advice from all the others, i.e., $a^{-i}=\left\{a_{t}^{ij}|j=1,\cdots \;,n,j\neq i\right\}$. At time *t* in the Boosting Phase, defining the probability of triggering the action advising from agent *j* for agent *i* as $pB_{t}^{ij}$, all the advice for agent *i* at time *t* can be defined as follows:


(9) $$\begin{array}{l} K_{t}^{i}=\left\{pB_{t}^{ij}a_{t}^{ij}|j=1,\cdots \;,n,j\neq i\right\}, \end{array}$$

Moreover, the knowledge at time *t* in the Boosting Phase, denoted by $K_{t}^{B}$, can be written as follows:


(10) $$\begin{array}{l} K_{t}^{B}=\left\{K_{t}^{i}|i=1,\cdots \;,n\right\}=\left\{a^{-i}|i=1,\cdots \;,n\right\}. \end{array}$$

With the action advice from the other agents, the focal agent can avoid some inappropriate decisions made by the moderate-level policy. At the same time, transferring action-based explicit knowledge can further improve the policy in the boosting phase.

### 3.3 When to transfer

Since the primary goal of KT-Hybrid for learning-from-scratch settings is to promote learning performance in the whole process, it is important to assess whether the knowledge should be transferred, i.e., when to transfer.

Mostly, the trigger conditions that enable knowledge transfer in the literature have the goal of transferring knowledge that can result in better task performance. However, we have to distinguish the different purposes between knowledge transfer in the multi-agent learning process and action demonstration in the process of task execution. When in the learning process, the goal of knowledge transfer should be to promote the **learning** performance rather than to enhance the correctness of some specific actions, which is especially true for the scenarios when all the agents learn simultaneously. Thus, it is necessary to re-consider what kind of knowledge is more beneficial to different learning phases in KT-Hybrid.

The Q value is a widely adopted metric to design trigger conditions (Silva and Costa, 2019). For the Igniting Phase, all of the agents are in the early learning stage. At this stage, conventional metrics such as Q values are not reliable since the networks have not been fully trained. At the same time, according to the explore–exploit balance, RL agents at this stage need to sufficiently explore the environment. With learning proceeds, the Q values calculated by the networks will be more accurate and reliable. Out of this consideration, in the Igniting Phase of KT-Hybrid, the probability of triggering knowledge transfer, i.e., sharing the current experience, for any agent *i* at time *t* is defined as $p_{t}^{i}$, which is calculated as follows:


(11) $$pI_{t}^{i}(Q_{t}^{i},{\bar{Q}}_{t}^{-i},\tau )=\left\{ \begin{array}{l} 0,f(Q_{t}^{i},\tau )\leq {\bar{Q}}_{t}^{-i}\\ 1,f(Q_{t}^{i},\tau )>{\bar{Q}}_{t}^{-i} \end{array}\right.$$

In Equation 11, ${\bar{Q}}_{t}^{-i}$ represents the mean value of the latest Q values received from the other agents, which can be written as follows:


(12) $$\begin{array}{l} {\bar{Q}}_{t}^{-i}=\frac{1}{\left(n-1\right)}\underset{j}{\sum }Q_{t}^{j},j\in N^{-1} \end{array}$$

in which $N^{-1}$ denotes the agents in the MARL system without agent *i*. Meanwhile, $f\left(Q_{t}^{i},\tau \right)$ in Equation 12 defines a scaling function inspired by the Sigmoid function, in which τ represents the learning steps that agent *i* has experienced. Formally, the scaling function can be calculated as follows:


(13) $$\begin{array}{l} f\left(Q_{t}^{i},\tau \right)=\frac{Q_{t}^{i}}{1+\exp\left(a-b\tau \right)},\tau \in {\mathbb{N}}^{+} \end{array}$$

where *a* and *b* are tuning hyper-parameters. Substituting Equations 12, 13 into Equation 11, we can find that when τ is small, i.e., when agent *i* has experienced little training, the sharing of experiences will be triggered as much as possible. When the value of τ increases, the agents will tend to share experiences with higher Q values. This matches our expectations for the balance between exploration and exploitation.

However, we should note that due to the trigger condition in the Igniting Phase (Equation 11) does not consider any specific observations, the agents can only get moderate-level policies from the overall perspective. Meanwhile, the agents will have different learning trajectories after the independent learning in the Igniting Phase, indicating they will be proficient in different states. Thus, building on the policies learned in the Igniting Phase, the agents need to further learn from the others' expertise in the Boosting Phase.

Inspired by Hou et al. (2021), we consider two metrics to evaluate the necessity of taking advice. The first one is success counts *l*_*i*_, the value of which counts the number of successful episodes that agent *i* has experienced. The other is the self-significance $h_{i}=Q_{t}^{i}/\max\left(\overset{i}{\underset{1}{Q}},\cdots \;,Q_{t}^{i}\right)$, which evaluates the significance of an action advice to the advisor. Given the above definitions, we define the advice from agent *j*, i.e., *a*^*ij*^, will only be qualified for agent *i* to choose when satisfying the following condition:


(14) $$\{\begin{array}{ll} l_{i} & <l_{j}\\ h_{i} & <h_{j} \end{array}$$

By the condition shown in Equation 14, only advice provided by agents with better overall performance that may have higher potential returns will be considered.

However, since an agent is allowed to take only one action at a time, we need to further design a merging module to resolve the conflicts among the qualified actions from different peers. Inspired by the multi-objective evolutionary algorithms, a ranking score *R*^*j*^ for each qualified action *a*^*ij*^ can be given as follows:


(15) $$\begin{array}{l} R_{j}={\hat{l}}_{j}\cdot h_{j}, \end{array}$$

in which ${\hat{l}}_{j}=l_{j}/\max\left(l_{1},\cdots \;,l_{n}\right)$ is the regulated success counts.

Given Equation 15, we can finally choose the action with the highest *R*_*j*_ as the final advice among the qualified candidates in the Boosting Phase.

As for the extra computational load in calculating the trigger conditions, this part of computation mainly involves three parts, which are the calculation of *l*_*i*_, *h*_*i*_, and *R*_*j*_, respectively. For the calculation of *l*_*i*_, it only needs to maintain a counting number of successful tasks. For *h*_*i*_ and *R*_*j*_, they need to find the maximum of a list and do multiplication once. All of these calculations only need simple algebraic operators. Note that although the computations are continually conducted, they have the same computing frequency of the policy network, i.e., for each agent, the trigger-condition-related calculations will only be conducted once after this agent makes a decision using the corresponding neural network. Compared with the computational load brought by the forward propagation, which consists of thousands of algebraic calculations such as multiplication, the extra computational load brought by the trigger conditions can be omitted. In addition, the computation cost of the trigger conditions in our proposed KT-Hybrid is comparable to many previous studies such as AdHocTD and AdHocVisit by Silva et al. (2017), eTL by Hou et al. (2017), and MeTL-ES by Wang et al. (2022). Thus, the extra computational load brought by the trigger conditions of KT-Hybrid is tolerable.

Another critical issue is how to determine the timing to shift the learning phase from the Igniting Phase to the Boosting Phase, which means we need a metric to determine whether the learning process is in the early or later stage. There are several potential principles in designing this shift scheme, including making the agents keep transferring knowledge in the whole learning process, preserving a longer Igniting Phase for less communicational cost, or letting the Boosting Phase intervene as early as possible for more efficient transfer. In this study, we take a straightforward shifting scheme by setting a fixed learning episode threshold *E* to divide the early and later learning stages. The learning process will be taken as the early learning stage before *E* episodes have been experienced, in which period the Igniting Phase will be triggered. Then, in the later learning stage, the Boost Phase will be used for the following learning process. The sensitivity of *E* will be tested later in Section 4. In addition, while the design of shifting schemes holds promise for exploring the potential of KT-Hybrid, delving into this aspect is currently beyond the scope of this study.

Now, we can provide the time complexity of the proposed KT-Hybrid. In deep RL, the main computational load lies in the decision-making (forward propagation) and learning (backpropagation) processes, both of which are related to the size of the network. In the following analysis, all the agents are assumed to use the same neural networks. Since the computational load of calculating the trigger conditions is much smaller than the neural network-related calculations, according to Equations 11–15; here, we omit this part and take the corresponding computational load as a constant $C_{t}$.

Assuming there are *n* agents in the MARL system, each of which conducts one round of both decision-making and training at each time step on average, the total amount of computation of the system can be formulated as follows:


(16) $$\begin{array}{l} c_{total}=\beta \cdot c_{I}+\left(1-\beta \right)\cdot c_{B}+C_{t}, \end{array}$$

in which *c*_*I*_ and *c*_*B*_ are the total computational load of the Igniting Phase and the Boosting Phase, respectively. β is a binary indicator showcasing the current phase. When the number of experienced episodes is less than *E*, we have β = 1, indicating the Igniting Phase is triggered; on the contrary, we have β = 0 in the Boosting Phase.

Denoting the computational load of one decision-making process of each agent as *c*_*f*_ and one backpropagation as *c*_*b*_, in the Igniting Phase, the total computational load of the system in a step can be written as


(17) $$\begin{array}{l} c_{I}=n\cdot c_{f}+n\cdot c_{b}=C\cdot n, \end{array}$$

where C is a constant.

For the Boosting Phase in which KT-Hybrid performs action advising, assuming the probability for each agent to ask for advice is *p*_*ask*_ and a probability *p*_*ans*_ for agents received the inquiries to provide advice to the advisees, and for each step, the total computational load of *n* learning agents can be given as follows:


(18) $$\begin{array}{l} c_{B}=n\cdot c_{f}+n\cdot c_{b}+p_{ask}\cdot n\cdot p_{ans}\cdot (n-1)\cdot c_{f}\\ \;=C_{1}\cdot n^{2}+C_{2}\cdot n, \end{array}$$

where $C_{1}$ and $C_{2}$ are constants.

Substituting Equations 17, 18 into Equation 16, the full computational load of the *n*-agent system in one step can be given as follows:


(19) $$\begin{array}{l} c_{total}=\beta \cdot (C\cdot n)+(1-\beta )\cdot (C_{1}\cdot n^{2}+C_{2}\cdot n)+C_{t}\\ \;=(1-\beta )\cdot C_{1}\cdot n^{2}+(C_{2}-\beta \cdot C_{2}+\beta \cdot C)\cdot n+C_{t}. \end{array}$$

Therefore, the time complexity of the proposed KT-Hybrid is *O*(*n*^2^). It is also noted that although the proposed KT-Hybrid shares the same time complexity with KT-AA methods, the computational cost of KT-Hybrid is less due to the Igniting Phase.

### 3.4 How to use the received knowledge

Having detailed the format and transfer timing of the knowledge, this subsection will introduce how to integrate the transferred knowledge into the learning process.

Non-stationarity is one of the most important issues to handle in the independent MARL processes. To prevent the learning performance from being affected by the non-stationarity brought by the simultaneous learning of multiple agents, a common solution is to disable the experience replay buffer (Palmer et al., 2018). Except for this, techniques such as synchronized learning have also been developed, which, however, also work at the cost of data efficiency. Meanwhile, these approaches can only reduce the influence of non-stationarity to some extent rather than fully remove it.

Therefore, in the Igniting Phase, we ignore the non-stationarity issue and learn with both the shared experiences, i.e., $K_{t}^{I}$, and the self-experienced ones, both of which are stored in a replay buffer D. The primary reason for neglecting the non-stationarity in this phase is to ensure data efficiency for better exploration of the environment, which helps to promote the policies as expected for the Igniting Phase. In addition, since the policies of the agents vary widely from random ones in the Igniting Phase, it should be difficult to significantly reduce the effect of non-stationarity. Out of consideration of data efficiency, the loss function for learning in the Igniting Phase can be written as follows:


(20) $$\begin{array}{l} L_{t}^{I}\left(\theta_{t}\right)={𝔼}_{o,a,r,o^{\prime }\sim D}\left[{\left(y-Q\left(o,a;\theta_{t}\right)\right)}^{2}\right]. \end{array}$$

In the Boosting Phase, the policies grow to a relatively stable level, which differs a lot from previous policies. Thus, it is important to avoid the non-stationarity brought by outdated experiences. Therefore, to further boost the performance, we only train the agents with the latest transitions in the Boosting Phase, i.e.,


(21) $$\begin{array}{l} L_{t}^{B}\left(\theta_{t}\right)={𝔼}_{o_{t},a_{t},r_{t},o_{t+1}}\left[{\left(y-Q\left(o_{t},a_{t};\theta_{t}\right)\right)}^{2}\right]. \end{array}$$

## 4 Empirical studies

This section provides simulation results to validate the effectiveness of the proposed KT-Hybrid.

### 4.1 Settings

**Figure 2:**
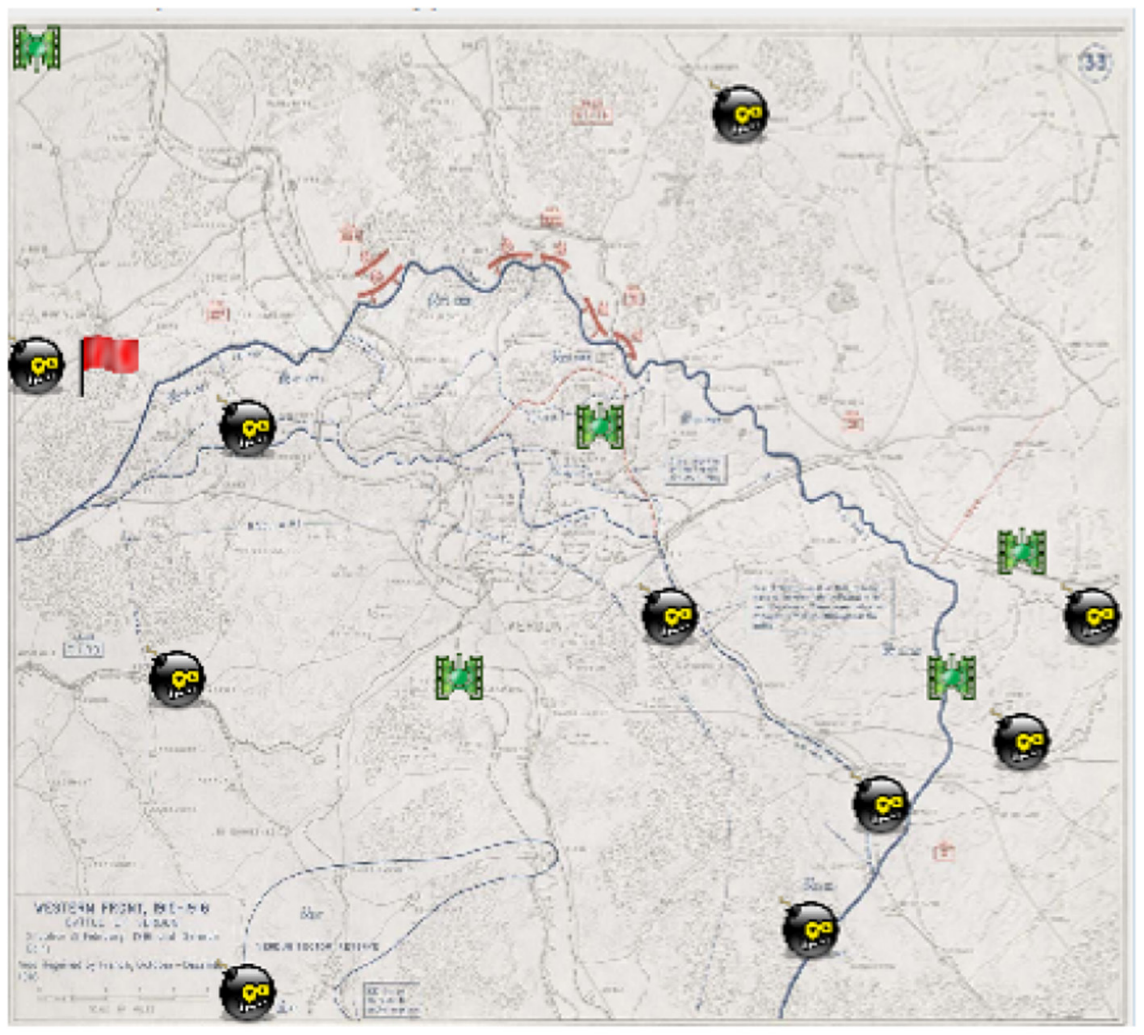
Snapshot of MNT environment.

To evaluate the effectiveness of the proposed KT-Hybrid, we compare the performance of KT-Hybrid in two scenarios. The first one is the Minefield Navigation Tasks (MNT) environment, and the results of KT-Hybrid are compared with MeTL-ES (Wang et al., 2022), eTL (Hou et al., 2021), and I-DQN (an independent MARL algorithm without knowledge transfer) (Tampuu et al., 2017), which represent state-of-the-art experience-sharing-based method, classical action advising method, and independent MARL without knowledge transfer, respectively. These knowledge transfer algorithms were also tested on MNT in their studies, so it is fair to compare these algorithms on MNT. Typically, the MNT environment includes moving agents, static “mines,” and a target in a grid world. The goal of the agents is to learn policies that can navigate them to the target position without collisions with each other or the mines. A typical snapshot of MNT environment is shown in Figure 2. Please refer to the Reference (Hou et al., 2021) and (Wang et al., 2022) for details of MNT.

In this section, we set the map of MNT as 16 × 16. The network of each learning agent is a fully connected two-layer multi-layer perceptron with 36 neurons in each layer. The learning rate is set to be 0.5. ϵ-greedy is utilized in the training process, the value of which anneals linearly from 0.5 to 0.005. Hyper-parameters *a* and *b* in Equation 13 are set to 5 and 0.001, respectively. The agents will be trained for 100,000 episodes, and the maximum length of each episode in MNT is set to 30 steps. For KT-Hybrid, the switch between the Igniting Phase and the Boosting Phase occurs after 10,000 episodes.

The second testing environment is the Half Field Offense (HFO) environment (Hausknecht et al., 2016), which was used as the testbed for AdHocVisit and AdHocTD (Silva et al., 2017). Moreover, we compare the performance of KT-Hybrid with AdHocVisit and AdHocTD for fairness.

**Figure 3:**
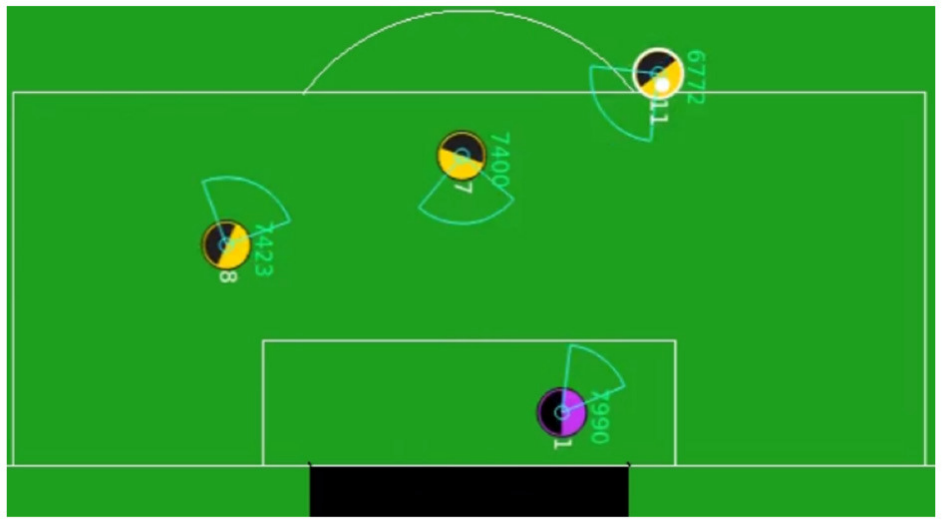
Snapshot of HFO environment.

Figure 3 depicts a snapshot of the HFO environment, including three agents learning from scratch, trying to score goals. Moreover, there is a goalkeeper with the Helios policy, which is from the 2012 RoboCup 2D champion team. To achieve fair comparison, we use the same environmental setting, learning parameters of AdHocTD and AdHocVisit, and the Helios policy with the study by Silva et al. (2017). For KT-Hybrid, the learning process shifts from the Igniting Phase to the Boosting Phase after 500 episodes.

### 4.2 Performance comparison

In this subsection, we first provide a comparison of the results in the MNT environment with 3 agents and 5 mines to validate the advantage of the proposed KT-Hybrid. All the results are generated by 30 independent runs.

**Figure 4:**
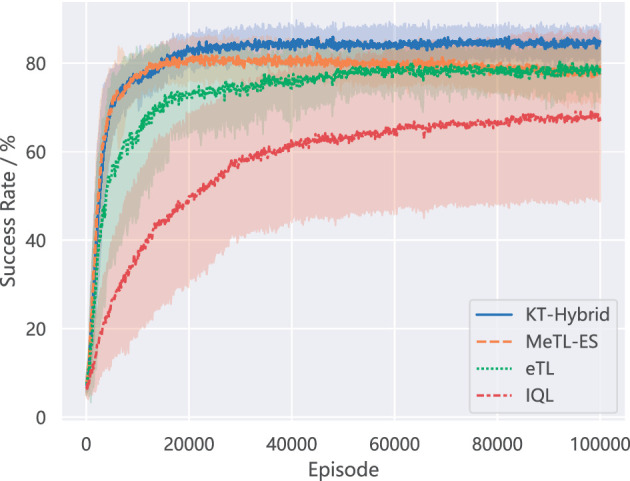
Comparision of success rate on MNT.

Figure 4 shows the learning performance of the proposed KT-Hybrid and the other baseline methods. The lines plot how the average success rate changes in the learning process, while the shadows depict one standard deviation. It is obvious that the proposed KT-Hybrid outperforms the other methods in the success rate of the task.

If we compare the results of KT-Hybrid with those of MeTL-ES, which is based on experience sharing, we will notice that although they both share a fast promotion in the learning process (Igniting Phase for KT-Hybrid), they behave differently later. With the learning proceeds, the line of MeTL-ES drops slightly from the best performance. While for the KT-Hybrid, the success rate keeps going up in the Boosting Phase. This difference matches our expectations when designing KT-Hybrid in Section 3, indicating that the proposed KT-Hybrid can achieve better performance by integrating experience-sharing-based and action-advising-based knowledge transfer approaches in the two-phase structure.

**Figure 5:**
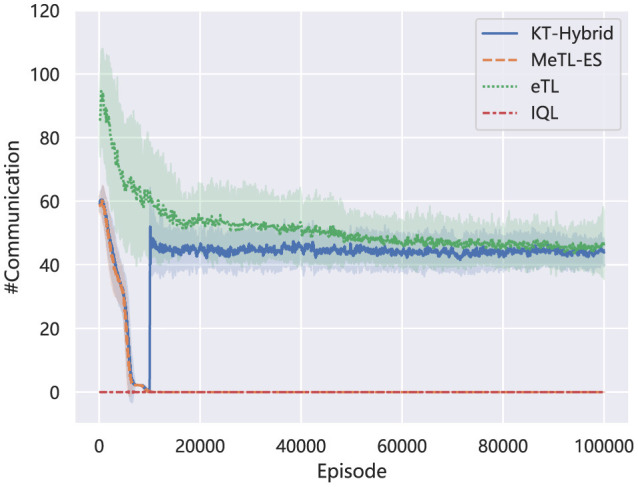
Comparision of the number of communication on MNT.

Figure 5 describes how the average times of communication vary in the learning process. In the Igniting Phase, the communication fluctuates similarly with the KT-ES method MeTL-ES, dropping rapidly to a very low level. This matches the trigger condition in this phase (Equations 11–13). To be specific, the threshold of the trigger condition in the Igniting Phase is calculated by Equation 12, which averages the latest received Q values from the other agents. As learning proceeds, the averaged Q value, i.e., ${\bar{Q}}_{t}^{-i}$, will converge to the mean value of the (sub)optimal Q values of each agent, which means that it will be harder for the agents to find a better Q value to trigger the experience sharing, according to Equation 11. When the learning process shifts to the Boosting Phase, the communication will be triggered by the Equations 14, 15, focusing on specific decision-making processes of all the agents. This enhances communication around the KT-AA level, which shows a sudden rise.

Combining the results in Figures 4, 5, we can find that the proposed KT-Hybrid can get significant and rapid performance enhancement with a moderate-level communication in the Igniting Phase. While for the Boosting Phase, although the algorithm needs more sufficient communication like all the action advising methods, it is still less than eTL. This phenomenon benefits from the two-phase architecture of KT-Hybrid. After the short Igniting Phase, the overall decision-making ability of the policies with KT-Hybrid is notably higher than that with eTL, as shown in Figure 4. This results in a higher starting point for the Boosting Phase than agents with eTL at the same stage. Consequently, agents with KT-Hybrid will need less advice than eTL to accomplish the task. This negative correlation between the number of communications and the success rate also indicates the rationality of the trigger conditions provided in Section 3.3.

Moreover, these results also indicate the superiority of using KT-Hybrid in real-world applications. With the help of KT-Hybrid, the users can get moderate-level policies in a very short time with limited communication in the Igniting Phase, which means that the agents can form a basic ability to solve certain tasks. Then, the users can decide how much extra training is needed in the Boosting Phase via systematical consideration of the performance and the training cost.

To further validate the superiority of KT-Hybrid, we also provide the comparison results of KT-Hybrid with AdHocTD and AdHocVisit on the HFO platform.

**Figure 6:**
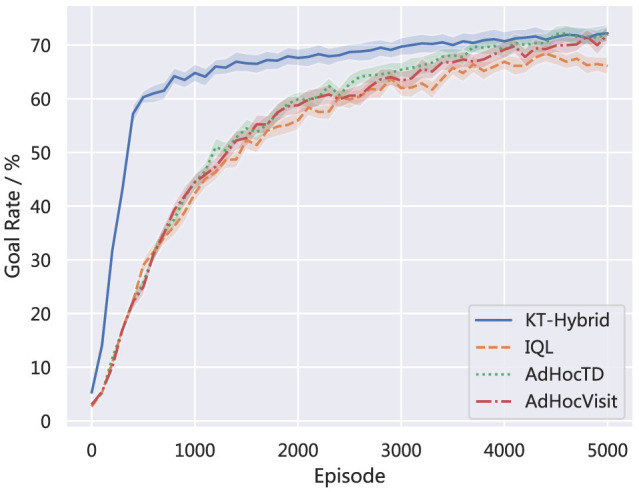
Comparision of the goal rate on HFO.

Figure 6 demonstrates the learning performance of the proposed KT-Hybrid with the other three baselines, which are IQL, AdHocTD, and AdHocVisit. The lines show the average rate of goal with learning proceeds. We can find that in HFO, our proposed KT-Hybrid still outperforms the baselines.

### 4.3 Validations in different scenarios

To make the results more convincing, in this subsection, we further compare the learning results with the aforementioned knowledge transfer algorithms in different experimental scenarios. Due to the limitation of space, from now on, we will only show the results on MNT.

**Figure 7:**
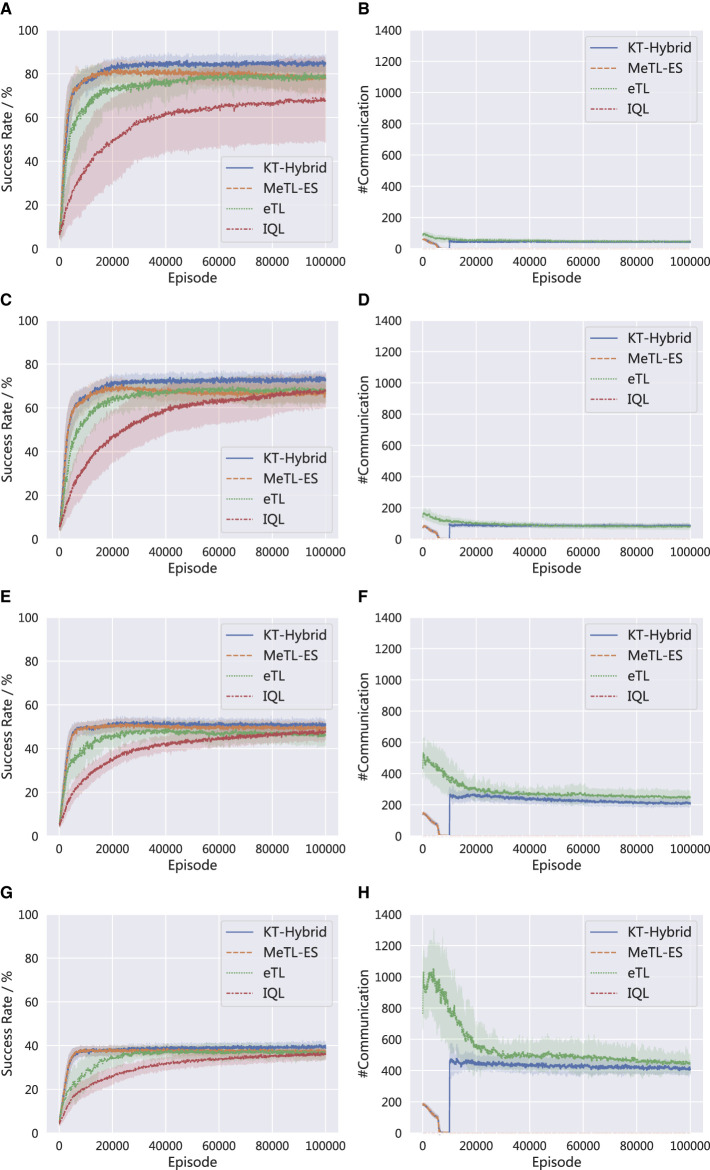
Performance comparison in different scenarios. **(A)** Success rate in 3a5m. **(B)** Number of communications in 3a5m. **(C)** Success rate in 5a10m. **(D)** Number of communications in 5a10m. **(E)** Success rate in 10a5m. **(F)** Number of communications in 10a5m. **(G)** Success rate in 15a3m. **(H)** Number of communications in 15a3m.

Figure 7 compares the success rates and the number of communications in different scenarios. In specific, the results are generated in MNT tasks with (a) & (b): 3 agents and 5 mines (3a5m), (c) & (d): 5 agents and 10 mines (5a10m), (e) & (f) 10 agents and 5 mines (10a5m), and (g) & (h): 15 agents and 3 mines (15a3m). It is noted that as the number of agents grows, the overall success rate decreases, and the number of communication increases. These trends are reasonable because as the scale grows, the tasks tend to become more complex, leading to an obvious performance decay. At the same time, the number of communications is enlarged since there are more agents involved in the knowledge transfer. Nevertheless, the superiority of KT-Hybrid remains in these scenarios, and the curve trends of KT-Hybrid are aligned with the results and discussions in Section 4.2.

Table 1 compares the average success rate of the different algorithms in different scenarios, which also indicates the advantage of KT-Hybrid. The bold values mark the best performance.

**Table 1:** Average success rate of the different knowledge transfer approaches in different scenarios.

**Scenario**	**KT-Hybrid**	**MeTL-ES**	**eTL**	**I-DQN**
3a5m	**81.03 %**	77.09 %	73.45 %	57.39 %
5a10m	**69.41 %**	65.45 %	63.60 %	55.29 %
10a5m	**49.46 %**	48.58 %	44.94 %	39.87 %
15a3m	**37.70 %**	36.62 %	34.45 %	30.31 %

### 4.4 Sensitivity analysis to the phase shifting time

To further validate the proposed KT-Hybrid, we have also added experiments on different settings of the phase shifting time. Specifically, we provide the KT-Hybrid with *E*= 5,000, 10,000, 20,000, and 50,000.

**Figure 8:**
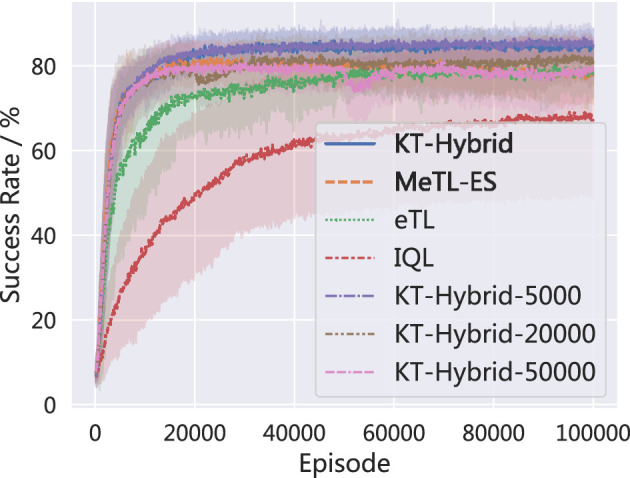
Performance comparison of different phase shifting time.

The success rates are shown in Figure 8. Note that the KT-Hybrid, with *E*= 10,000 is the setting we use in Section 4.2, denoted as KT-Hybrid in Figure 8 for consistency. It is clear that all the settings we provide can effectively enhance the learning speed, compared with MeTL-ES, eTL, and IQL. At the same time, we should note that the earlier we shift the phase, the better the performance we have, although the difference is only approximately 5%. Given the results presented in Figure 8, we can find that the KT-Hybrid is not very sensitive to the phase-shifting time *E*, but we still need to conduct more research on how to choose the phase-shifting time in the future.

## 5 Conclusion

This study proposed a novel knowledge transfer method for independent MARL, namely, KT-Hybrid. The proposed KT-Hybrid integrates the widely adopted KT-AA and the recently proposed KT-ES into a two-phase architecture. For the early learning stage, agents with KT-Hybrid conduct the Igniting Phase, trying to leverage the high data efficiency of KT-ES to achieve fast promotion of the decision-making ability via experience sharing. Then, based on the moderate-level policies obtained by the Igniting Phase, the following Boosting Phase tries to further enhance the performance via transferring actions. Empirical studies on several MNT scenarios show that the performance of the proposed KT-Hybrid matches our expectations in design and outperforms the baselines.

However, KT-Hybrid exhibits certain limitations. A primary concern lies in the manually adjusted shift timing between the Igniting Phase and the Boosting Phase, which necessitates reliance on the user's domain expertise. To address this challenge, several paths for future research emerge. First, a more comprehensive investigation on the influence of hyperparameters through theoretical analysis is desired. Such an approach can offer insights into the underlying mechanisms governing the performance of KT-Hybrid. Second, the exploration of automated tuning methods, leveraging cutting-edge intelligent decision-making techniques such as evolutionary algorithms, holds promise for enhancing the efficacy of KT-Hybrid. Furthermore, the development of a data-driven metric for evaluating the optimal phase selection within the algorithm is also promising. This metric, hopefully, could further enhance the adaptability and robustness of KT-Hybrid.

## Data availability statement

The raw data supporting the conclusions of this article will be provided on request.

## Author contributions

FL: Writing – original draft, Validation, Software, Resources, Methodology, Investigation, Funding acquisition, Formal analysis, Data curation, Conceptualization. DL: Writing – review & editing, Visualization, Validation, Resources, Data curation. JG: Writing – review & editing, Supervision, Project administration, Methodology, Investigation, Conceptualization.
